# Supplementary material for: Grocery shopping as an outcome measure: A scoping review
Source: PLoS One. 2025 May 20;20(5):e0324711. doi: 10.1371/journal.pone.0324711 (PMC12091886; doi:10.1371/journal.pone.0324711)
Supplement: Attachment — Submitted filename: Response to Reviewers.docx [file pone.0324711.s003.docx]

**Response to Reviewers**

We thank the reviewers and editor for your thoughtful appraisal and comments.

Each reviewer-suggested change is listed in black, and our response to each suggestion is listed immediately below in blue.

Reviewer #1: 
1. The studies reviewed vary significantly in methodology, population, and assessment tools, making direct comparisons difficult. While this is a limitation of scoping reviews, it should be explicitly acknowledged. The study does not conduct a meta-analysis or any quantitative synthesis, which limits the ability to draw strong numerical conclusions about trends across studies.

We completely agree with your statement. We stated in the Study outcomes section previously “Although the heterogeneous nature of the studies included in this scoping review did not allow for meta-analysis, we did investigate the themes of the major findings of each study” We acknowledge that this is not a complete assessment of the limitations. We have now added the following sentence to the limitations section. “The studies reviewed varied significantly in methodology, population, and assessment tools, which limits the ability to make direct comparisons between studies and renders meta-analysis or quantitative synthesis impractical.“

2. Including a summary of key findings in percentages would enhance readability.

Thank you for this suggestion. We have now included percentages through the methods section along with the study counts for each category discussed.

3. Supplemental Table 1 provides extracted study data, ensuring transparency. If possible, include a table summarizing key study findings numerically to improve data interpretation.

Thank you for this suggestion. Also in alignment with a comment from Reviewer 3, we have addressed this by including the number of studies for each result category in the pie chart, which hopefully will improve the ability for readers to quantify the subgroups that we were able to discuss in the results and discussion sections of this paper.

Reviewer #2:

1. In the results, I couldn´t find references to the studies of Ogourtsova et al., 2018; Kizony et al., 2010 and Chang et al., 2022. in the study population section (lines 158-172). In the assessment delivery method section, I couldn´t find references to the studies of McGuire et al., 2006 and Bosley et al., 2004. Since these papers meet the inclusion criteria and were described in the country of origin section I think they should be described and cited in all other results sections.

Thank you for catching this oversight! We absolutely agree that these studies should also be included in these sections. They have now been included in each section. In explanation, we inadvertently left stroke and the 3 associated citations out of the study population section. Citation errors were made in the assessment delivery, which have now been corrected. 1) McGuire 2006 is referenced for “IADL Profile” instead of Bier et al., 2013. In the same section, Bier et al., was accurately referenced for testing in a real-world grocery store in the previous draft. 2) Bosley et al., 2004 is now referenced in the statement of “only asked a specific question related to grocery shopping ability or independence” instead of Kiosses et al., 2005. Kiosses et al., 2005 is referenced correctly for the Multi-Level Assessment IADLS scale.

2. In the introduction, the sentence "Both the SF-36 and PROMIS measures have been validated across a wide range of populations, including those experiencing aging, schizophrenia, and neurodegenerative diseases.[5, 6]" (lines 68-70) refers to the '36-Item Short Form Health Survey questionnaire' as SF-36, I think it is best to use the full name in this first reference and maintain the acronym in the next ones.

Thank you for bringing this to our attention. This has now been corrected in the previous sentence where they first appear.

3. In this same sentence, the author mentions populations experiencing aging, schizophrenia, and neurodegenerative diseases, however, the work of Hill et al., 2001 and Shields et al., 2008 (references 5 and 6) describes patients with chronic obstructive pulmonary disease and adults with Down syndrome, respectively. I think it would be better if the authors could add references to populations experiencing aging, schizophrenia, and neurodegenerative diseases.

Thank you for bringing this oversight to our attention. This is now updated with references for these specific populations.

4. Also, in line 70, and several other lines, the reference number is placed after the comma, the authors should carefully scan the paper to uniform this.
Thank you for catching this inconsistency. Reference numbers are now placed before all commas and periods.

5. In the introduction, the sentence "Verbal memory tests such as these have 79 been shown to be sensitive in the ability to detect differences in cognitive function in 80 conditions such as Alzheimer’s disease[9] and mild traumatic brain injury [9]." (lines 78-80) mention populations experiencing Alzheimer’s disease and mild traumatic brain injury, but the work of Leitner et al., 2019 describes a heterogeneous sample of people with traumatic brain injury. It would be better if the authors could add a reference directly related to populations experiencing Alzheimer’s too.

Thank you for catching this referencing error. This is now corrected with an appropriate reference to a study involving those affected by Alzheimer’s disease.

5. The introduction section has excellent content, but, if the authors can group the related concepts and cut some words, it will be even better.
We appreciate the comment and have reviewed the introduction in the context of the rest of the manuscript. We believe that the format of the current manuscript is important to demonstrate the multi-layered paradigm that is key to understanding dual and multi-tasking in an ecologically valid environment. If we group concepts together, the information does not reflect the complex nature of what we have been trying to understand. We hope that helps clarify.

6. In the discussion, the sentence "Of these 15 studies, nine emphasized the relationship between cognitive function and grocery shopping ability and how cognitive functioning in this population relates to being able to function independently in complex real-world tasks.[34-38, 41, 42, 56, 57]" (lines 221-224), I understand that by "these 15 studies" the authors mean the studies with individuals with psychiatric disorders. However, since the number 15 was used to refer to these studies only in the results section, maybe it's best to repeat something like "studies with individuals with psychiatric disorders" to make it clear.

Thank you for this suggestion. The statement has been changed to “Of the studies including individuals with psychiatric disorders, “

7. I found the sentence "These studies provide evidence that mobility devices (e.g., scooters) increase a person’s independence in grocery shopping and reported grocery shopping ability at baseline and two months following total hip arthroplasty is related to pain and one-year functional outcomes." (lines 284-287) a little bit hard to understand. I think it will be better If it can be divided into two small sentences.
Thank you for this suggestion. The sentence has been broken up into two sentences: “These studies provide evidence that mobility devices (e.g., scooters) increase a person’s independence in grocery shopping. These studies also report findings that reported grocery shopping ability at baseline and two months following total hip arthroplasty is related to pain and one-year functional outcomes.“

8. The sentence "Finally, and most markedly, there is a lack of ecologically valid functional assessments for people on the spectrum of ADRD, even though the definition of dementia includes the loss of ability to perform ADLs/IADLs." (lines 322-324) Maybe the authors should use 'Alzheimer's disease and related dementias' before using ADRD to make the paper more readable to people who are not from the field.

Thank you for the suggestion. This has been changed accordingly.

Reviewer #3:

1. In Figure 2, in the study population, instead of adult, write healthy adult.
Thank you for bringing this to our attention. This is now corrected.

2. In the discussion, line 213, I think the correct word is neurocognitive instead of neurodevelopmental.
Thank you for catching this typographical error. This is now corrected.

3. In the study population, line 165 mentions a study that uses only a population with schizophrenia; but, in the discussion, line 221 mentions schizophrenia or schizoaffective disorder, it is not clear since they are two different disorders.
Thank you for bringing this to our attention. Some of the included studies examined both schizophrenia and schizoaffective disorder in the same study. This statement has been revised to “schizophrenia or schizoaffective disorder[]”.

4. In figure 2, the study population includes a study with people who have suffered a stroke, however, this is not mentioned in the results or in the discussion.
We apologize; this is now included in the results in the “Study populations” section.

5. For a better understanding of the graphs in figure 2, it would be good to include the number of studies within the graph.

Thank you for this suggestion. We have now added the number of studies in each wedge of each pie chart.

While revising your submission, please upload your figure files to the Preflight Analysis and Conversion Engine (PACE) digital diagnostic tool, [https://pacev2.apexcovantage.com/](https://nam12.safelinks.protection.outlook.com/?url=https%3A%2F%2Ftrack.editorialmanager.com%2FCL0%2Fhttps%3A%252F%252Fpacev2.apexcovantage.com%252F%2F1%2F010f0196279fa135-8dd9911d-01db-4d02-a9fd-bdabf123826c-000000%2FkSFETI9s1hNidF45WXDywPUVUSBt7lO5d6snvau2SmA%3D206&data=05%7C02%7Ckeith.cole%40vumc.org%7C4417fdb5932d404db03008dd7961c3ab%7Cef57503014244ed8b83c12c533d879ab%7C0%7C0%7C638800183329752676%7CUnknown%7CTWFpbGZsb3d8eyJFbXB0eU1hcGkiOnRydWUsIlYiOiIwLjAuMDAwMCIsIlAiOiJXaW4zMiIsIkFOIjoiTWFpbCIsIldUIjoyfQ%3D%3D%7C0%7C%7C%7C&sdata=hFSpUFFWb17X1W6N8dRIfzrgRfdqxpV%2Bf2dr3ic%2BPMc%3D&reserved=0). PACE helps ensure that figures meet PLOS requirements. To use PACE, you must first register as a user. Registration is free. Then, login and navigate to the UPLOAD tab, where you will find detailed instructions on how to use the tool. If you encounter any issues or have any questions when using PACE, please email PLOS at [figures@plos.org](mailto:figures@plos.org). Please note that Supporting Information files do not need this step.
